# Toll-Like Receptor-Mediated Cardiac Injury during Experimental Sepsis

**DOI:** 10.1155/2020/6051983

**Published:** 2020-01-10

**Authors:** Ina Lackner, Birte Weber, Shinjini Chakraborty, Sonja Braumüller, Markus Huber-Lang, Florian Gebhard, Miriam Kalbitz

**Affiliations:** ^1^Department of Traumatology, Hand, Plastic, and Reconstructive Surgery, Center of Surgery, University of Ulm, 89081 Ulm, Germany; ^2^Institute of Clinical and Experimental Trauma Immunology, University of Ulm, 89081 Ulm, Germany

## Abstract

Sepsis is associated with global cardiac dysfunction and with high mortality rate. The development of septic cardiomyopathy is due to complex interactions of damage-associated molecular patters, cytokines, and complement activation products. The aim of this study was to define the effects of sepsis on cardiac structure, gap junction, and tight junction (TJ) proteins. Sepsis was induced by cecal ligation and puncture in male C57BL/6 mice. After a period of 24 h, the expression of cardiac structure, gap junction, and TJ proteins was determined. Murine HL-1 cells were stimulated with LPS, and mRNA expression of cardiac structure and gap junction proteins, intracellular reactive oxygen species, and troponin I release was analyzed. Furthermore, pyrogenic receptor subtype 7 (P2X7) expression and troponin I release of human cardiomyocytes (iPS) were determined after LPS exposure. *In vivo*, protein expression of connexin43 and *α*-actinin was decreased after the onset of polymicrobial sepsis, whereas in HL-1 cells, mRNA expression of *connexin43*, *α-actinin*, and *desmin* was increased in the presence of LPS. Expression of TJ proteins was not affected *in vivo* during sepsis. Although the presence of LPS and nigericin resulted in a significant troponin I release from HL-1 cells. Sepsis affected cardiac structure and gap junction proteins in mice, potentially contributing to compromised cardiac function.

## 1. Introduction

Severe sepsis is associated with a high lethality rate, cardiac dysfunction, and heart failure in rodents as well as in humans [1, 2]. In humans, myocardial depression during sepsis is described as a global systolic and diastolic dysfunction, including right ventricular (RV) and left ventricular (LV) malfunction, and is characterized by increased morbidity and mortality [3, 4]. During experimental sepsis, cardiac output, LV stroke volume, and LV ejection fraction decreased in mice, which reflect the cardiodepressive effects of sepsis [5]. This cardiac dysfunction is also called cardiomyopathy of sepsis [6]. Of note, mice with LV dilatation showed improved cardiovascular performance and increased survival in CLP sepsis [7]. Patients suffering from septic cardiomyopathy showed enhanced plasma levels of cardiac troponin (cTn), correlating with an increased mortality rate [8, 9]. During sepsis, various cardiodepressive biomarkers such as tumor necrosis factor (TNF), interleukin- (IL-) 1*β*, and complement factor 5a (C5a) are released [10–13]. In addition, damage-associated molecular patterns (DAMPs), such as extracellular histones and the high mobility group box-1 protein (HMGB1), were enhanced during sepsis [14, 15]. The complement activation product C5a plays a dominant role in sepsis and is associated with the development of septic cardiomyopathy [6]. C5a acts via two G-protein-coupled C5a receptors (C5aR1 and C5aR2), triggering a massive increase of reactive oxygen species (ROS) and intracellular calcium [Ca^2+^i]. Enhanced ROS was associated with cardiac remodelling, reduced LV function, and contractile dysfunction [16, 17]. Increased [Ca^2+^i] affected the homeostasis and the electrophysiological functions of cardiomyocytes (CMs) [5]. By altering and disturbing their action potentials, C5a induced defects in CM contractility [5].

In human hearts, sepsis induced a redistribution of the gap junction protein connexin43 (Cx43) from the intercalated discs to the lateral membranes of the CMs [18]. Further, reduced Cx43 expression was associated with structural damage of the intercalated discs and with the loss of structural integrity in CMs [19]. In rat hearts, the Cx43 mRNA expression decreased likewise dramatically and rapidly within 2 h after injection of lipopolysaccharide (LPS) [19]. Moreover, Cx43 expression was also decreased in the presence of TNF, suggesting that circulating, cardiodepressive cytokines are able to modulate Cx43 expression during sepsis in rodents as well as in septic patients [20]. However, little is known about the effects of sepsis on the expression and distribution of other cardiac gap and tight junction proteins and cardiac structure. In previous experiments, our group showed that severe trauma affected cardiac structure and the localization of gap junction proteins [21, 22]. Furthermore, we demonstrated that multiple trauma in pigs altered the expression of cardiac structure proteins, such as *α*-actinin and desmin. Furthermore, translocation of the gap junction proteins as accessed by both, zonula occludens-1 (ZO-1) and Cx43 translocation to the cytosol, has been shown after multiple trauma in pigs [22]. This translocation has been shown to lead to a loss of mechanical and electrical coupling of the CMs, finally resulting in cardiac dysfunction [22]. Further, Cx43 translocation was also observed in rats after blunt chest trauma [21], again indicating disrupted cardiac electrical coupling, resulting in arrhythmias [21, 23]. In this study we investigated the effects of septic conditions on cardiac structure, gap junction, and tight junction proteins.

## 2. Materials and Methods

### 2.1. *In Vivo* Experiments

#### 2.1.1. Animals and Anaesthesia

All procedures were performed after obtaining approval from the University of Ulm Committee on the Use and Care of Animals (approval number 988). 8-12-week-old C57Bl6 male mice weighing 25-30 g had access to food and water *ad libitum.*

#### 2.1.2. Experimental Sepsis

Littermate mice were randomly assigned in sham and CLP groups, with *n* = 5 mice per group. Mice were anaesthetized with 2.5% sevoflurane (Sevorane Abbott, Germany) and 97.5% oxygen throughout the procedure and were given 0.03 mg/kg buprenorphine by subcutaneous injection for analgesia. CLP was induced as previously described [24]. An abdominal midline incision was given after shaving the region. To induce midgrade sepsis, a ligation was applied halfway between the ileocecal valve and the ending of the cecum. A 21 G needle was used to make a through-and-through puncture of the cecum. A minimal amount of bowel content was extruded and the cecum was relocated. The abdominal incision was closed in layers using 4–0 sutures (Ethilon, Ethicon GmbH, Norderstedt, Germany). Fluid resuscitation was performed by 1 ml of 0.9% saline (Jonosteril) applied subcutaneously in the nuchal region. For the sham procedure, the same steps were followed except ligating and puncturing the cecum. Every 6 hours, the mice were monitored and buprenorphine is injected subcutaneously. Animals were allowed free access to water and food before and after experimental procedures. 24 hours after surgery, the mice were sacrificed. Hearts were obtained and paraffin embedded for further analysis.

### 2.2. *In Vitro* Experiments

For the *in vitro* experiments, the murine cardiac muscle cell line (HL-1 cells) [25] (Sigma Aldrich, St. Louis, MO, USA) and human cardiomyocytes (iPS) (Cellular Dynamics, Madison, WI, USA) were used. Murine HL-1 cells were cultured in an HL-1 expansion medium at 37°C in an atmosphere of 5% CO_2_. Human cardiomyocytes (iPS) were cultured for 10 days in a maintenance medium (Cellular Dynamics, Madison, WI, USA) at 37°C in an atmosphere of 7% CO_2_.

Next, HL-1 cells were treated with 20 *μ*g/ml LPS and the human cardiomyocytes with 10 *μ*g/ml LPS for 6 h at 37°C. For the determination of troponin I elevation of cells in the presence of LPS with either ATP or nigericin, the cells were treated for 5 h with LPS with the abovementioned concentrations and for one further hour either with 1 mM ATP (Sigma Aldrich, St. Louis, MO, USA) or with 10 *μ*M nigericin (Sigma Aldrich, St. Louis, MO, USA). The control groups were incubated in cell culture media without any supplements.

### 2.3. RNA Isolation

RNA isolation from cell lysates was performed by using the ISOLATE II RNA Mini Kit (Meridian Bioscience, Cincinnati, OH, USA). The remaining DNA was digested by DNaseI (Meridian Bioscience, Cincinnati, OH, USA).

### 2.4. Quantitative Reverse Transcribed Polymerase Chain Reaction (RT-qPCR)

The respective RNA samples were reverse transcribed in cDNA using SuperScript™ IV VILO™ MasterMix (Invitrogen, Carlsbad, CA, USA). For quantitative PCR, the PowerUp™ SYBR™ Green Master Mix (Applied Biosystems, Waltham, MA, USA) was used. All procedures were performed according to the manufacturers' instructions. For qPCR, the QuantStudio 3 system (Applied Biosystems, Waltham, MA, USA) was utilized. Quantitative mRNA expression of mouse *α-actinin* (forward: 5′-AACCTGGCCATGGAAATAGCA-3′, reverse: 5′-ATCGGGTTTGGGAGTGTTGA-3′), mouse *connexin40* (forward: 5′-GAGCAAATAACAGTGGGCAGT-3′, reverse: 5′-CGAGCCTTCTGCTTCCTTTCC-3′), mouse *connexin43* (forward: 5′-GGCCACAGGTGAGACCATTA-3′, reverse: 5′-CGGCCATCGTTGTTCTTGTC-3′), mouse *connexin45* (forward: 5′-TTGCCAAAATGGAGCATGGC-3′, reverse: 5′-TTCCGTTTCTTCCAGAGCCC-3′), mouse *desmin* (forward: 5′-CTCGGATATCACACCCAGCC-3′, reverse: 5′-CACAAAGGGGTGATCGGTGA-3′), mouse *troponinI* (forward: 5′-GATGCGGCTGGGGAACC-3′, reverse: 5′-ACTTTTTCTTGGCGTGTGGC-3′), and human *pyrogenic receptor (P2X7)* (forward: 5′-CACACCAAGGTGAAGGGGAT-3′, reverse: 5′-GGTGTAGTCTGCGGTGTCAA-3′) was examined and calculated by the cycle threshold method *Δ*ΔCt. Respective genes were normalised using housekeeping gene *glutaraldehyde-phosphate dehydrogenase (GAPDH)* for mouse (forward: 5′-CTTCAACAGCAACTCCCACTCTTCC-3′, reverse: 5′-GGTGGTCCAGGGTTTCTTACTCC-3′) and for human *GAPDH* (forward: 5′-TCTCTGCTCCTCCTGTTCGAC-3′, reverse: 5′-CCAATACGACCAAATCCGTTGA-3′). Results are presented as mean fold change.

### 2.5. Reactive Oxygen Species (ROS)

After LPS treatment, cells were incubated for further 30 min with 5 *μ*M CellROX® Deep Red Reagent (Life Technologies, Carlsbad, CA, USA) at 37°C and 5% CO_2_. Afterwards, cells were fixed with 4% formaldehyde and the cell nuclei were stained with Hoechst. Cells were evaluated by fluorescence microscopy using an Axio Imager M.2 microscope and the Zeiss ZEN 2.3 software (Zeiss, Jena, Germany). A relative amount of reactive oxygen species was determined by ImageJ software [26] (National Institutes of Health, Bethesda, MD, USA).

### 2.6. Troponin I ELISA

Supernatant fluids from HL-1 cells from human cardiomyocytes (iPS) were collected after LPS treatment, and troponin I was determined by using either ultrasensitive murine cardiac TroponinI ELISA (Life Diagnostics, West Chester, PA, USA) or the Cardiac Troponin I Human SimpleStep ELISA® Kit (Abcam, Cambridge, UK). All procedures were performed according to the manufacturers' instructions.

### 2.7. Immunofluorescence Staining of Cardiomyocytes (IF)

Cells were fixed with 4% formaldehyde for 15 min at RT and permeabilized with 0.3% Triton-X for 10 min at RT. Unspecific binding sites were blocked with 10% goat serum, and specific antigen detection was performed by incubating cells with the respective primary antibodies for ryanodine receptor 1 (RyR1) (Abcam, Cambridge, UK), desmin (GeneTex, Irvine, CA, USA), and troponin I (Abcam, Cambridge, UK) for overnight at 4°C. Specific antibody binding was detected by using either AlexaFluor647-labelled (Jackson ImmunoResearch, West Grove, PA, USA) or rhodamine-labelled (Jackson ImmunoResearch, West Grove, PA, USA) secondary antibodies. Cell nuclei were stained with Hoechst. Fluorescence was investigated by fluorescence microscopy using an Axio Imager M.2 microscope and the Zeiss ZEN 2.3 software (Zeiss, Jena, Germany). Fluorescence intensities were evaluated using ImageJ software (National Institutes of Health, Bethesda, MD, USA). Values are illustrated as mean fluorescence intensity.

### 2.8. General Procedure for Immunofluorescence (IF), Immunohistochemical (IHC), and PAS Staining of Paraffin-Embedded Left Ventricles

Paraffin sections of the respective left ventricles were deparaffinised and rehydrated. Antigen unmasking was performed in 10 mM citrate buffer (pH 6) at 100°C. Tissues' own peroxidase was blocked by incubating sections in 3% H_2_O_2_ solution for 15 min at RT. Unspecific binding sites were blocked with 10% goat serum.

### 2.9. Immunofluorescence Staining of Left Ventricles (IF)

Specific antigen binding was performed by incubating sections with the respective primary antibodies for *α*-actinin (GeneTex, Irvine, CA, USA), desmin (GeneTex, Irvine, CA, USA), troponin I (Abcam, Cambridge, UK), connexin43 (Cell Signalling Technology, Cambridge, UK), zonula occludens-1 (Bioss, Woburn, MA, USA), claudin-18 (ThermoFisher, Waltham, MA, USA), and occludin (Bioss, Woburn, MA, USA) overnight at 4°C. Specific antibody binding was detected by using either AlexaFluor488-labelled (Jackson ImmunoResearch, West Grove, PA, USA), rhodamine-labelled (Jackson ImmunoResearch, West Grove, PA, USA), or AlexaFluor647-labelled (Jackson ImmunoResearch, West Grove, PA, USA) secondary antibodies. Cell nuclei were counterstained with Hoechst. Sections were analysed by fluorescence microscopy by using an Axio Imager M.2 microscope and the Zeiss ZEN 2.3 software (Zeiss, Jena, Germany). Fluorescence intensities as well as the amount of apoptotic cells were evaluated using ImageJ software (National Institutes of Health, Bethesda, MD, USA). Results are presented as mean fluorescence intensity.

### 2.10. Immunohistochemistry (IHC) and PAS Staining

Specific antigen binding was determined by incubating sections with the respective primary antibodies for Cx43 (Cell Signalling Technology, Cambridge, UK), C3aR (Bioss, Woburn, MA, USA), and IL-1*β* (Abcam, Cambridge, UK) overnight at 4°C. A biotinylated IgG (Life Technologies, Carlsbad, CA, USA) was used as secondary antibody. Signal amplification was performed by using the VECTASTAIN® ABC Kit (Vector Laboratories, Burlingame, CA, USA), and signal was obtained by developing sections with the VECTOR® NovaRED™ Peroxidase Substrate Kit (Vector Laboratories, Burlingame, CA, USA). Cell nuclei were counterstained in haematoxylin. Sections were investigated by bright-field microscopy using an Axio Imager M.2 microscope and the Zeiss ZEN 2.3 software (Zeiss, Jena, Germany). Results are presented as mean pixel density. PAS staining was performed using a PAS-staining kit (Merck Millipore, Darmstadt, Germany). Signal density was measured using an Axio Imager M.2 microscope and the Zeiss ZEN 2.3 software (Zeiss, Jena, Germany). Results are presented as mean density.

### 2.11. Statistical Analysis

All values are expressed as mean ± SEM. Data were analysed by one-way ANOVA, followed by Dunnett's or Tukey's multiple comparison test. *p* ≤ 0.05 was considered statistically significant. GraphPad Prism 7.0 software was used for statistical analysis (GraphPad Software, Incorporated, San Diego, CA, USA).

## 3. Results

### 3.1. Expression of C3aR and IL-1*β* and Cardiac Glycogen Storage during Sepsis In Vivo

**Figure 1:**
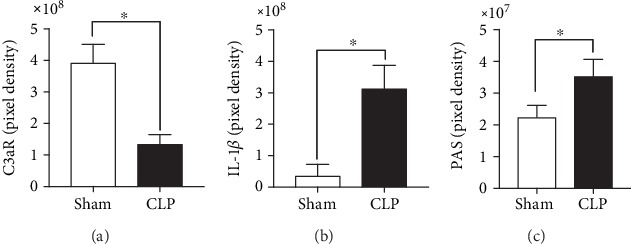
Cardiac physiological, immunological, and damage-associated alterations *in vivo* in left ventricles of CLP mice: mean pixel density of complement factor 3a receptor (C3aR) (a), interleukin-1*β* (IL-1*β*) (b), and glycogen storage in left ventricles in pixel density (c). Mice received sham treatment (white bars) or CLP treatment (black bars) for 24 h. Left ventricles were collected 24 h after treatment. *n* = 5 animals per group; ^∗^*p* < 0.05. All values are expressed as mean ± SEM.

In order to determine the inflammatory condition during CLP sepsis *in vivo*, the expression of the complement factor 3a receptor (C3aR) as well as the expression of interleukin- (IL-) 1*β* was determined in the left ventricles by microscopy. In the left ventricles, the expression of C3aR decreased significantly, whereas the expression of IL-1*β* increased during sepsis compared to sham-treated animals (Figures 1(a) and 1(b)). Furthermore, the cardiac glycogen storage was determined by PAS staining. Cardiac glycogen storage increased significantly in CLP mice (Figure 1(c)).

### 3.2. Cardiac Structural Alterations during Sepsis In Vivo

**Figure 2:**
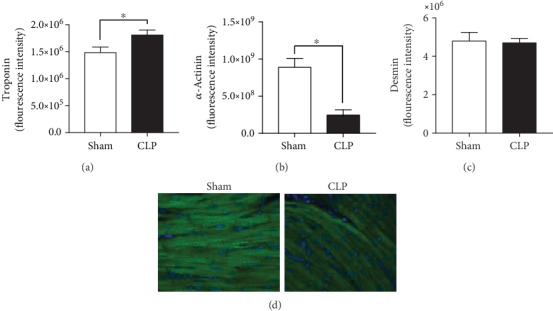
Cardiac structural alterations *in vivo* in left ventricles of CLP mice: fluorescence intensities of troponin I (a), *α*-actinin (b), and desmin (c) and representative picture of *α*-actinin in left ventricles (d). Mice received either sham treatment (white bars) or CLP treatment (black bars) for 24 h. Left ventricles were collected 24 h after treatment. *n* = 5 animals per group; ^∗^*p* < 0.05. All values are expressed as mean ± SEM.

Since the animals showed enhanced inflammation, which was probably mediated via NLRP3-inflammasome signalling and via complement activation, the effects of CLP sepsis on cardiac structure proteins were determined in left ventricles by microscopy. In the left ventricles, troponin I expression in CLP mice increased slightly but significantly compared to that in sham-treated animals (Figure 2(a)), whereas the *α*-actinin expression decreased in these mice during sepsis (Figures 2(b) and 2(d)). No differences were seen in the desmin expression during sepsis (Figure 2(c)). Moreover, CLP mice showed severe internal bleeding in the left ventricles (Supplemental Figure 1).

### 3.3. Cardiac Gap and Tight Junction Protein Expression during Sepsis In Vivo

**Figure 3:**
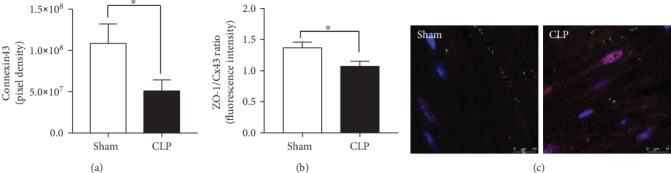
Alterations of cardiac gap junction proteins *in vivo* in left ventricles of CLP mice: expression of connexin43 in pixel density (a), ratio of the colocalization of zonula occludens-1/connexin43 (b), and representative picture of colocalization of zonula occludens-1/connexin43 (c). Mice received either sham treatment (white bars) or CLP treatment (black bars) for 24 h. Left ventricles were collected 24 h after treatment. *n* = 5 animals per group; ^∗^*p* < 0.05. All values are expressed as mean ± SEM.

Since the expression of specific cardiac structure proteins was altered during sepsis *in vivo*, the expression of cardiac tight and gap junctions was investigated by microscopy. In the left ventricles, the expression of Cx43 decreased during sepsis (Figure 3(a)). Moreover, the ratio of co-located proteins zonula occludens-1 (ZO-1)/Cx43 changed (Figures 3(b) and 3(c)), and both proteins were cotranslocated from the intercalated discs into the cytosol of CMs. The expression of claudin-18 and occludin did not change during sepsis (data not shown) in left ventricular tissue.

### 3.4. Functional and Structural Alterations in HL-1 Cells in Presence of LPS In Vitro

**Figure 4:**
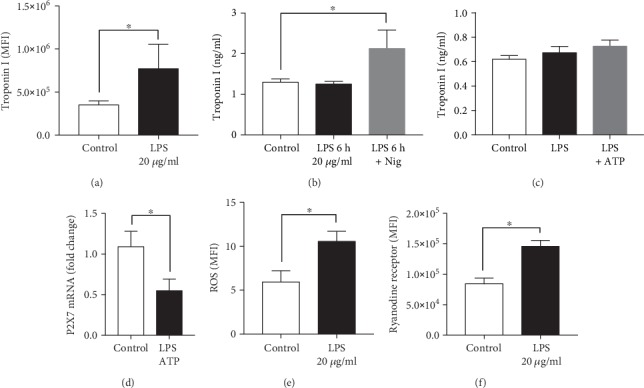
Effects of LPS on murine HL-cells. Fluorescence intensity of troponin I expression of HL-1 cells (a). Troponin I elevation in supernatant fluids from HL-1 cells (b) and from human cardiomyocytes (c). mRNA expression of pyrogenic receptor (P2X7) in human cardiomyocytes (d). HL-1 cells were treated with 20 *μ*g/ml LPS and human cardiomyocytes (iPS) with 10 *μ*g/ml LPS for 6 h (black bars). Additional treatment included treatment with 20 *μ*g/ml or 10 *μ*g/ml LPS for 5 h, respectively, and for one further hour with either 1 mM ATP or 1 *μ*M nigericin (grey bars). Control groups were incubated in a cell culture medium without any supplements for 6 h (white bars). Amount of cellular reactive oxygen species (ROS) (e). Fluorescence intensity of ryanodine receptor 1 (RyR1) (f). *n* = 6 per group; ^∗^*p* < 0.05. All values are expressed as mean ± SEM.

During CLP sepsis, the expression of cardiac structure and gap junction proteins was altered, which was probably mediated via inflammatory mediators. Therefore, the effects of LPS on murine HL-1 cells were investigated *in vitro*. The cellular troponin I expression and the release of troponin I in supernatant fluids were investigated in order to determine the damage of the cells. Furthermore, the troponin I release was investigated when the cells were treated additionally with ATP or nigericin in order to determine an P2X7-dependent troponin I release. The protein expression of troponin I increased significantly in HL-1 cells in the presence of LPS (Figure 4(a)). Troponin I concentration increased significantly in supernatant fluids from HL-1 cells in presence of LPS and nigericin compared to control (Figure 4(b)) as well as in supernatant from human CMs, in the presence of LPS and ATP (Figure 4(c)). Moreover, mRNA expression of *pyrogenic receptor (P2X7)* decreased in human CMs treated with LPS and ATP (Figure 4(d)).

Furthermore, the amount of cytosolic ROS was determined. The amount of cytosolic ROS increased significantly in HL-1 cells in the presence of LPS compared to control (Figure 4(e)). Since ROS is able to alter the channel open probability of the ryanodine receptor 1 (RyR1), the expression of this receptor was determined by fluorescence microscopy. The protein expression of the RyR1 significantly increased in HL-1 cells in presence of LPS, compared to control group (Figure 4(f)).

**Figure 5:**
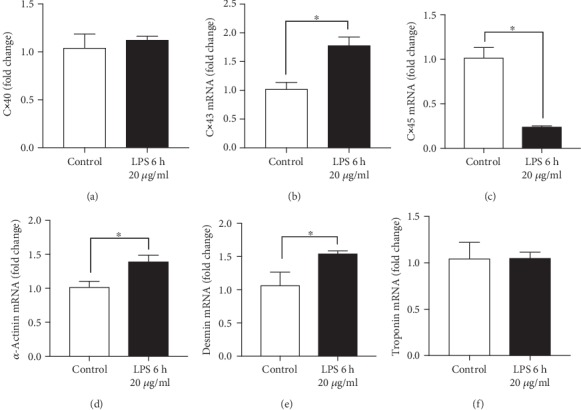
Gene expression in HL-1 cells in presence of LPS. mRNA expression of connexin 40 (Cx40) (a), connexin43 (Cx43) (b), connexin 45 (Cx45) (c), *α*-actinin (d), desmin (e), and troponin I (f). Results are presented as mean fold change. HL-1 cells were treated with 20 *μ*g/ml LPS for 6 h (black bars). The control group was incubated in a cell culture medium without any supplements (white bars). *n* = 6 per each group; ^∗^*p* < 0.05. All values are expressed as mean ± SEM.

Since the expression of cardiac structure and gap junction proteins was altered during CLP sepsis *in vivo*, the effects of LPS on mRNA expression of specific structure and gap junction proteins were analyzed *in vitro*. The mRNA expression of *connexin40* (Figure 5(a)) and *troponin I* (Figure 5(f)) did not change in presence of LPS, in contrast the mRNA expression of *connexin43* (Figure 5(b)), *α-actinin* (Figure 5(d), and *desmin* (Figure 5(e)) increased slightly but significantly. The mRNA expression of *connexin45* decreased in presence of LPS (Figure 5(c)).

## 4. Discussion

In mice with CLP-induced sepsis, troponin I expression increased significantly in the left ventricles. This upregulation of the troponin I was probably due to compensation of cardiac troponin, since cTn was excessively released during sepsis in humans [27, 28] and during experimental sepsis in rodents [29]. Release of troponin I has been associated with an increased mortality risk in septic patients [8, 9, 30]. The cTn release into circulation during sepsis might be due to myocardial membrane leakage or to a release in a cytokine-dependent manner [31]. In the present study, troponin I expression in HL-1 cells significantly increased in the presence of LPS. Accordingly, in supernatants from HL-1 cells as well as from human CMs, the troponin I concentration increased in the presence of LPS. Interestingly, this troponin I release was further increased in the presence of either additional ATP or nigericin, indicating for a toll-like receptor- (TLR-)/pyrogenic receptor- (P2X7-) mediated/NLRP3-inflammasome-dependent release of troponin I from CMs. The decreased *P2X7* mRNA expression strengthened this assumption. In earlier studies, we demonstrated increased NLRP3 and IL-1*β* expression in cardiomyocytes during sepsis in mice [32] and substantial release of IL-1*β* from cardiomyocytes in presence of LPS [32] and ATP or nigericin. NLRP3 inflammasome activation has been linked to complement activation [32]. In the present study, the expression of the IL-1*β* increased in left ventricles during sepsis as well, which was already demonstrated in hearts of septic rats and mice 8 h after CLP [32, 33].

Excessive IL-1*β* release might be due to the complement-dependent activation of the NLRP3 inflammasome. This activation leads to enhanced release of cardiodepressive IL-1*β*, resulting in disturbance of myocardial function and cardiac damage [32, 34].

In earlier studies, the C5aR was upregulated in hearts during experimental sepsis [35]. Absence of either C5aR1 or C5aR2 was protective for heart function during sepsis [5, 36]. Furthermore, the activation of complement system during sepsis resulted in the generation of C3a and inactivated C3desArg [35, 37]. C3a has been linked to multiple organ failure during sepsis [38]. The role of the C3a receptor in the heart during sepsis is still unknown. In bronchial epithelial and smooth muscle cells in mice, C3aR has been reported to be increased during conditions of endotoxemia [39], whereas plasma concentration of IL-1*β* was significantly elevated in C3aR^−/−^ mice following LPS application, indicating that C3aR act as an anti-inflammatory receptor by attenuating LPS-induced proinflammatory cytokine production [40]. On the other hand, C3aR^−/−^ mice were more susceptible to shock after LPS administration [40]. In the present study, in cardiac tissue, C3aR expression was significantly reduced whereas IL-1*β* expression was increased. In isolated peripheral blood mononuclear cells (PBMC), C3a acted as an anti-inflammatory factor by suppressing LPS-induced secretion of IL-1*β*, TNF, and IL-6 [41, 42]. In this study, we demonstrated for the first time that complement activation during sepsis is associated with reduced C3aR expression and simultaneous increase of IL-1*β* expression.

NLRP3^−/−^ mice had ameliorated cardiac dysfunction during sepsis [32]. The use of a selective NOX2 inhibitor prevented the release of cytosolic and mitochondrial ROS levels and the release of IL-1*β*. The tertiary structure of NLRP3 containing a highly conserved disulphide bond connecting the pyrin domain, which is very sensitive to altered redox states [43].

In our study, in presence of LPS, the amount of ROS increased in HL-1 cells, which might be due to the activation of NADPH oxidase Nox2 [5, 44]. Moreover, CLP- and endotoxin-induced sepsis enhanced the mRNA expression of NADPH oxidase Nox1 *in vivo*, also leading to increased production of ROS [45]. Moreover, LPS induced ROS production in isolated rat CMs *in vitro*, which seemed to be mediated via the toll-like receptor 4/NADPH oxidase Nox4 (TLR4-NOX4) pathway [46].

In previous studies, we further showed that CMs of rats after CLP exhibited enhanced cytosolic ROS between 8 h and 48 h during sepsis. Moreover, excessive ROS altered and modified proteins involved in calcium handling and electrical coupling of CMs, resulting in cardiac and contractile dysfunction [16, 17]. The alterations of the calcium regulatory proteins sarcoplasmic/endoplasmic reticulum calcium ATPase (SERCA2a) and sodium/calcium exchanger (NCX) during CLP were demonstrated previously in mice and were associated with impaired cardiac contractility and relaxation, leading finally to severe heart failure [5, 47–49]. The left ventricular expression as well as the activity of SERCA2a and NCX were clearly decreased during both CLP- and endotoxin-induced sepsis [50–52].

In the present study, the expression of the ryanodine receptor (RyR) 1 was increased after exposure of HL-1 cells to LPS *in vitro*. In a recently published work, TLR4 has been shown to mediate septic cardiomyopathy by an increased RyR leak [53]. Oxidative modification of RyR resulted in conformational change and alteration of open probability [54, 55]. Oxidative stress therefore has been demonstrated to be a major protagonist of sarcoplasmic reticulum Ca^2+^ leak in heart failure [56, 57].

TLR signaling has further been linked to changes in glycolytic metabolism in various cell types such as dendritic cells [58] and plays a critical role in host defense and inflammation. Thereby, a metabolic switch was induced from oxidative phosphorylation to aerobic glycolysis in different immune cells such as macrophages and leucocytes, which is also known as the Warburg effect [58–63]. So far, it was demonstrated that enhanced serum lactate levels due to increased glycolysis correlate positively with the outcome in septic patients, leading to early hyperlactatemia during sepsis [64–66]. Further, lactate was shown to activate innate immunity and inflammatory response via TLR signaling and via inflammasomes [67]. In addition, enhanced cardiac glycolysis was demonstrated in mice 6 h after CLP and was associated with impaired cardiac function during sepsis [68]. In the present study, cardiac glycogen deposits increased significantly 24 h after CLP sepsis, which was shown previously in rodents with sepsis as well as in pigs after multiple trauma [22, 69]. Increased deposits of glycogen, as well as increased glucose uptake and increased glucose transporter 4 (GLUT4) expression, were also described previously in mice 48 h after CLP and were associated with myocardial hibernation of the CMs in the septic heart [69]. This specific metabolic condition of the CMs is characterized by the upregulation of GLUT4, which prevents cardiac cell damage and LV dysfunction in the injured hypoxic myocardium by enhanced glucose uptake [69–73].

Compromised calcium handling and metabolic alterations in the heart during sepsis have been linked to TLR signaling. In intestinal epithelial cells, TLR2 has been shown to enhance barrier resistance by apical redistribution of ZO-1 [74]. TLR2 stimulation has been demonstrated to amplify gap junctional intercellular communication (GJIC) in acute intestinal epithelia cell (IEC) barrier injury by transcriptional regulation and by posttranslational modification of Cx43 [75]. TLR2 stimulation upregulated Cx43 mRNA expression in the intestinal epithelial and in parallel, Cx43-P_2_ protein isoform redistributed to the plasma membrane, suggesting enhanced incorporation of hemichannels in gap junction plaques [76]. However, TLR2 activation in alveolar epithelial cells has been shown to disrupt GJIC by induction of c-Src-mediated tyrosine phosphorylation of Cx43 [77]. This might be protective for the host from bacterial dissemination through gap junctions [78]. In the heart, Cx43 expression was significantly decreased during sepsis, which is in accordance to earlier findings in humans post mortem [18]. The reduction of the Cx43 protein might be induced by enhanced circulating TNF concentrations, which arise during sepsis, reducing the electrical and chemical coupling of cardiomyocytes [19, 79]. Thereby, the conductance of Cx43 may be reduced by phosphorylation by the Ca^2+^-dependent protein kinase C (PKC) [80]. Moreover, in the present study, Cx43 was translocated from intercalated discs into the cytosol of the CMs (data not shown), which is in accordance to earlier studies in different trauma models, as well as in septic rodents and humans [18–22]. The translocation of the Cx43 was also demonstrated previously in ischemic as well as in nonischemic cardiac injury [81–83]. Gap junction endocytosis of Cx43 was associated with disruption of electrical coupling of CMs, resulting finally in arrhythmia and cardiac dysfunction [23, 84, 85]. Further, the two gap junction proteins ZO-1/Cx43 were colocalized into the cytosol during sepsis, which is in accordance with earlier studies in multiple injured pigs where this phenomenon was associated with impaired cardiac function after trauma [22].

In contrast to findings in the gut [86] or in the lungs [87], the expression of the tight junction proteins was not affected during sepsis in the heart (data not shown). In other inflammatory conditions and locations such as chronic inflammatory pain, tight junction proteins of the blood brain barrier responded to inflammation by up- and downregulation of their protein expression [88]. In cultured alveolar epithelial cells isolated from rats, claudin-18 and occludin were significantly reduced during sepsis, whereas ZO-1 was not significantly affected [89]. After administration of LPS in mice, ZO-1 expression in kidneys was increased after 6 h and reduced after 24 h, whereas ZO-1 mRNA expression varied but in opposite direction [90]. Therefore, the unchanged expression of tight junction proteins claudin-18 and occludin in the present study was probably due to the time point and has to be evaluated in more detail in future studies.

Further, cardiac structural proteins located in the Z-lines were investigated. *α*-Actinin, which is colocalized with L-type calcium channels and stabilizes the muscle contractile apparatus of cardiomyocytes, was reduced in left ventricles during sepsis. In vascular smooth muscle cells, *α*-actinin have been downregulated after LPS application in mice [91], which is in accordance to our findings. Loss of proteins associated with the sarcomeric skeleton such as *α*-actinin may contribute to cardiac dysfunction during sepsis. Moreover, mice showed cardiac injury and severe internal bleeding in left ventricles, which was associated with septic cardiomyopathy.

## 5. Conclusion

Taken together, our results suggest that TLR signaling is involved in cardiac redox signaling as well as calcium handling and energy metabolism during sepsis. Alterations in gap junction and Z-disc proteins during sepsis linked to TLRs. Therefore, therapeutic intervention addressing TLRs might be a promising approach to ameliorate cardiac dysfunction during sepsis.

## Data Availability

The RT-qPCR and protein expression data used to support the findings of this study are available from the corresponding author upon request.

## Supplementary Materials

Supplementary Materials Supplemental Figure 1: haematoxylin and eosin staining of the left ventricles of sham and CLP mice. Representative images were taken in 20x and 40x magnification. Mice received either sham treatment or CLP treatment for 24 h.

## References

[1] Fattahi F., Ward P. A. (2017). Complement and sepsis-induced heart dysfunction. *Molecular Immunology*.

[2] Russel J. A. (2008). The current management of septic shock. *Minerva Medica*.

[3] Antonucci E., Fiaccadori E., Donadello K., Taccone F. S., Franchi F., Scolletta S. (2014). Myocardial depression in sepsis: from pathogenesis to clinical manifestations and treatment. *Journal of Critical Care*.

[4] Vieillard-Baron A., Prin S., Chergui K., Dubourg O., Jardin F. (2003). Hemodynamic instability in sepsis: bedside assessment by Doppler echocardiography. *American Journal of Respiratory and Critical Care Medicine*.

[5] Kalbitz M., Fattahi F., Herron T. J. (2016). Complement destabilizes cardiomyocyte function in vivo after polymicrobial sepsis and in vitro. *Journal of Immunology*.

[6] Niederbichler A. D., Hoesel L. M., Westfall M. V. (2006). An essential role for complement C5a in the pathogenesis of septic cardiac dysfunction. *The Journal of Experimental Medicine*.

[7] Zanotti Cavazzoni S. L., Guglielmi M., Parrillo J. E., Walker T., Dellinger R. P., Hollenberg S. M. (2010). Ventricular dilation is associated with improved cardiovascular performance and survival in sepsis. *Chest*.

[8] Fromm R. E. (2007). Cardiac troponins in the intensive care unit: common causes of increased levels and interpretation. *Critical Care Medicine*.

[9] Gualandro D. M., Puelacher C., Mueller C. (2014). High-sensitivity cardiac troponin in acute conditions. *Current Opinion in Critical Care*.

[10] Atefi G., Zetoune F. S., Herron T. J. (2011). Complement dependency of cardiomyocyte release of mediators during sepsis. *The FASEB Journal*.

[11] Finkel M. S., Oddis C. V., Jacob T. D., Watkins S. C., Hattler B. G., Simmons R. L. (1992). Negative inotropic effects of cytokines on the heart mediated by nitric oxide. *Science*.

[12] Kumar A., Thota V., Dee L., Olson J., Uretz E., Parrillo J. E. (1996). Tumor necrosis factor alpha and interleukin 1beta are responsible for in vitro myocardial cell depression induced by human septic shock serum. *The Journal of Experimental Medicine*.

[13] Parrillo J. E., Burch C., Shelhamer J. H., Parker M. M., Natanson C., Schuette W. (1985). A circulating myocardial depressant substance in humans with septic shock. Septic shock patients with a reduced ejection fraction have a circulating factor that depresses in vitro myocardial cell performance. *The Journal of Clinical Investigation*.

[14] Denk S., Perl M., Huber-Lang M. (2012). Damage- and pathogen-associated molecular patterns and alarmins: keys to sepsis?. *European Surgical Research*.

[15] Seong S. Y., Matzinger P. (2004). Hydrophobicity: an ancient damage-associated molecular pattern that initiates innate immune responses. *Nature Reviews Immunology*.

[16] Tsutsui H., Kinugawa S., Matsushima S. (2011). Oxidative stress and heart failure. *American Journal of Physiology. Heart and Circulatory Physiology*.

[17] Zima A. V., Blatter L. A. (2006). Redox regulation of cardiac calcium channels and transporters. *Cardiovascular Research*.

[18] Takasu O., Gaut J. P., Watanabe E. (2013). Mechanisms of cardiac and renal dysfunction in patients dying of sepsis. *American Journal of Respiratory and Critical Care Medicine*.

[19] Celes M. R. N., Torres-Dueñas D., Alves-Filho J. C., Duarte D. B., Cunha F. Q., Rossi M. A. (2007). Reduction of gap and adherens junction proteins and intercalated disc structural remodeling in the hearts of mice submitted to severe cecal ligation and puncture sepsis. *Critical Care Medicine*.

[20] Fernandez-Cobo M., Gingalewski C., Drujan D., De Maio A. (1999). Downregulation of connexin 43 gene expression in rat heart during inflammation. The role of tumour necrosis factor. *Cytokine*.

[21] Kalbitz M., Amann E. M., Bosch B. (2017). Experimental blunt chest trauma-induced myocardial inflammation and alteration of gap-junction protein connexin 43. *PLoS One*.

[22] Kalbitz M., Schwarz S., Weber B. (2017). Cardiac Depression in Pigs after Multiple Trauma - Characterization of Posttraumatic Structural and Functional Alterations. *Scientific Reports*.

[23] Nakagami T., Tanaka H., Dai P. (2008). Generation of reentrant arrhythmias by dominant-negative inhibition of connexin 43 in rat cultured myocyte monolayers. *Cardiovascular Research*.

[24] Rittirsch D., Huber-Lang M. S., Flierl M. A., Ward P. A. (2009). Immunodesign of experimental sepsis by cecal ligation and puncture. *Nature Protocols*.

[25] Claycomb W. C., Lanson N. A., Stallworth B. S. (1998). HL-1 cells: a cardiac muscle cell line that contracts and retains phenotypic characteristics of the adult cardiomyocyte. *Proceedings of the National Academy of Sciences of the United States of America*.

[26] Schneider C. A., Rasband W. S., Eliceiri K. W. (2012). NIH image to ImageJ: 25 years of image analysis. *Nature Methods*.

[27] Mehta N. J., Khan I. A., Gupta V., Jani K., Gowda R. M., Smith P. R. (2004). Cardiac troponin I predicts myocardial dysfunction and adverse outcome in septic shock. *International Journal of Cardiology*.

[28] Røsjø H., Varpula M., Hagve T.-A. (2011). Circulating high sensitivity troponin T in severe sepsis and septic shock: distribution, associated factors, and relation to outcome. *Intensive Care Medicine*.

[29] Yousif N. G., Hadi N. R., Al-Amran F., Zigam Q. A. (2018). Cardioprotective effects of irbesartan in polymicrobial sepsis. *Herz*.

[30] Hamilton M. A., Toner A., Cecconi M. (2012). Troponin in critically ill patients. *Minerva Anestesiologica*.

[31] Altmann D. R., Korte W., Maeder M. T. (2010). Elevated cardiac troponin I in sepsis and septic shock: no evidence for thrombus associated myocardial necrosis. *PLoS One*.

[32] Kalbitz M., Fattahi F., Grailer J. J. (2016). Complement-induced activation of the cardiac NLRP3 inflammasome in sepsis. *The FASEB Journal*.

[33] Borge B. Å. S., Kalland K. H., Olsen S., Bletsa A., Berggreen E., Wiig H. (2009). Cytokines are produced locally by myocytes in rat skeletal muscle during endotoxemia. *American Journal of Physiology. Heart and Circulatory Physiology*.

[34] Cain B. S., Meldrum D. R., Dinarello C. A. (1999). Tumor necrosis factor-alpha and interleukin-1 beta synergistically depress human myocardial function. *Critical Care Medicine*.

[35] Ward P. A. (2008). Role of the complement in experimental sepsis. *Journal of Leukocyte Biology*.

[36] Kalbitz M., Grailer J. J., Fattahi F. (2015). Role of extracellular histones in the cardiomyopathy of sepsis. *The FASEB Journal*.

[37] Smedegard G., Cui L. X., Hugli T. E. (1989). Endotoxin-induced shock in the rat. A role for C5a. *The American Journal of Pathology*.

[38] Hack C. E., Nuijens J. H., Felt-Bersma R. J. F. (1989). Elevated plasma levels of the anaphylatoxins C3a and C4a are associated with a fatal outcome in sepsis. *The American Journal of Medicine*.

[39] Drouin S. M., Kildsgaard J., Haviland J. (2001). Expression of the complement anaphylatoxin C3a and C5a receptors on bronchial epithelial and smooth muscle cells in models of sepsis and asthma. *Journal of Immunology*.

[40] Kildsgaard J., Hollmann T. J., Matthews K. W., Bian K., Murad F., Wetsel R. A. (2000). Cutting edge: targeted disruption of the C3a receptor gene demonstrates a novel protective anti-inflammatory role for C3a in endotoxin-shock. *Journal of Immunology*.

[41] Takabayashi T., Vannier E., Burke J. F., Tompkins R. G., Gelfand J. A., Clark B. D. (1998). Both C3a and C3a(desArg) regulate interleukin-6 synthesis in human peripheral blood mononuclear cells. *The Journal of Infectious Diseases*.

[42] Takabayashi T., Vannier E., Clark B. D. (1996). A new biologic role for C3a and C3a desArg: regulation of TNF-alpha and IL-1 beta synthesis. *Journal of Immunology*.

[43] Bae J. Y., Park H. H. (2011). Crystal structure of NALP3 protein pyrin domain (PYD) and its implications in inflammasome assembly. *The Journal of Biological Chemistry*.

[44] Joseph L. C., Kokkinaki D., Valenti M. C. (2017). Inhibition of NADPH oxidase 2 (NOX2) prevents sepsis-induced cardiomyopathy by improving calcium handling and mitochondrial function. *JCI Insight*.

[45] Matsuno K., Iwata K., Matsumoto M. (2012). NOX1/NADPH oxidase is involved in endotoxin-induced cardiomyocyte apoptosis. *Free Radical Biology & Medicine*.

[46] Zhao H., Zhang M., Zhou F. (2016). Cinnamaldehyde ameliorates LPS-induced cardiac dysfunction via TLR4-NOX4 pathway: the regulation of autophagy and ROS production. *Journal of Molecular and Cellular Cardiology*.

[47] Arai M., Alpert N. R., MacLennan D. H., Barton P., Periasamy M. (1993). Alterations in sarcoplasmic reticulum gene expression in human heart failure. A possible mechanism for alterations in systolic and diastolic properties of the failing myocardium. *Circulation Research*.

[48] He H., Giordano F. J., Hilal-Dandan R. (1997). Overexpression of the rat sarcoplasmic reticulum Ca^2+^ ATPase gene in the heart of transgenic mice accelerates calcium transients and cardiac relaxation. *The Journal of Clinical Investigation*.

[49] Piacentino V., Weber C. R., Chen X. (2003). Cellular basis of abnormal calcium transients of failing human ventricular myocytes. *Circulation Research*.

[50] Hobai I. A., Buys E. S., Morse J. C. (2013). SERCA Cys674 sulphonylation and inhibition of L-type Ca2+ influx contribute to cardiac dysfunction in endotoxemic mice, independent of cGMP synthesis. *American Journal of Physiology. Heart and Circulatory Physiology*.

[51] Hobai I. A., Edgecomb J., LaBarge K., Colucci W. S. (2015). Dysregulation of intracellular calcium transporters in animal models of sepsis-induced cardiomyopathy. *Shock*.

[52] Wu J., Zhang J. Y., Gong Y., Li S. T. (2016). Slowed relaxation of diaphragm in septic rats is associated with reduced expression of sarco-endoplasmic reticulum CA^2+^ -ATPase genes *SERCA1* and *SERCA2*. *Muscle & Nerve*.

[53] Yang J., Zhang R., Jiang X. (2018). Toll-like receptor 4-induced ryanodine receptor 2 oxidation and sarcoplasmic reticulum Ca^(2+)^ leakage promote cardiac contractile dysfunction in sepsis. *The Journal of Biological Chemistry*.

[54] Anzai K., Ogawa K., Kuniyasu A., Ozawa T., Yamamoto H., Nakayama H. (1998). Effects of hydroxyl radical and sulfhydryl reagents on the open probability of the purified cardiac ryanodine receptor channel incorporated into planar lipid bilayers. *Biochemical and Biophysical Research Communications*.

[55] Xia R., Stangler T., Abramson J. J. (2000). Skeletal muscle ryanodine receptor is a redox sensor with a well defined redox potential that is sensitive to channel modulators. *The Journal of Biological Chemistry*.

[56] Mochizuki M., Yano M., Oda T. (2007). Scavenging free radicals by low-dose carvedilol prevents redox-dependent Ca2+ leak via stabilization of ryanodine receptor in heart failure. *Journal of the American College of Cardiology*.

[57] Yano M., Okuda S., Oda T. (2005). Correction of defective interdomain interaction within ryanodine receptor by antioxidant is a new therapeutic strategy against heart failure. *Circulation*.

[58] Krawczyk C. M., Holowka T., Sun J. (2010). Toll-like receptor-induced changes in glycolytic metabolism regulate dendritic cell activation. *Blood*.

[59] Cheng S. C., Joosten L. A., Netea M. G. (2014). The interplay between central metabolism and innate immune responses. *Cytokine & Growth Factor Reviews*.

[60] Cheng S. C., Scicluna B. P., Arts R. J. W. (2016). Broad defects in the energy metabolism of leukocytes underlie immunoparalysis in sepsis. *Nature Immunology*.

[61] Denk S., Neher M. D., Messerer D. A. C. (2017). Complement C5a functions as a master switch for the pH balance in neutrophils exerting fundamental immunometabolic effects. *Journal of Immunology*.

[62] O'Neill L. A., Pearce E. J. (2016). Immunometabolism governs dendritic cell and macrophage function. *The Journal of Experimental Medicine*.

[63] Vander Heiden M. G., Cantley L. C., Thompson C. B. (2009). Understanding the Warburg effect: the metabolic requirements of cell proliferation. *Science*.

[64] Chertoff J., Chisum M., Garcia B., Lascano J. (2015). Lactate kinetics in sepsis and septic shock: a review of the literature and rationale for further research. *Journal of Intensive Care*.

[65] Garcia-Alvarez M., Marik P., Bellomo R. (2014). Sepsis-associated hyperlactatemia. *Critical Care*.

[66] Mikkelsen M. E., Miltiades A. N., Gaieski D. F. (2009). Serum lactate is associated with mortality in severe sepsis independent of organ failure and shock. *Critical Care Medicine*.

[67] Samuvel D. J., Sundararaj K. P., Nareika A., Lopes-Virella M. F., Huang Y. (2009). Lactate boosts TLR4 signaling and NF-kappaB pathway-mediated gene transcription in macrophages via monocarboxylate transporters and MD-2 up-regulation. *Journal of Immunology*.

[68] Zheng Z., Ma H., Zhang X. (2017). Enhanced glycolytic metabolism contributes to cardiac dysfunction in polymicrobial sepsis. *The Journal of Infectious Diseases*.

[69] Levy R. J., Piel D. A., Acton P. D. (2005). Evidence of myocardial hibernation in the septic heart. *Critical Care Medicine*.

[70] Kirkeboen K. A. (1995). The hibernating myocardium. *Tidsskrift for den Norske Lægeforening*.

[71] McFalls E. O., Murad B., Haspel H. C., Marx D., Sikora J., Ward H. B. (2003). Myocardial glucose uptake after dobutamine stress in chronic hibernating swine myocardium. *Journal of Nuclear Cardiology*.

[72] Nishino Y., Miura T., Miki T. (2004). Ischemic preconditioning activates AMPK in a PKC-dependent manner and induces GLUT4 up-regulation in the late phase of cardioprotection. *Cardiovascular Research*.

[73] Tian R., Abel E. D. (2001). Responses of GLUT4-deficient hearts to ischemia underscore the importance of glycolysis. *Circulation*.

[74] Cario E., Gerken G., Podolsky D. K. (2004). Toll-like receptor 2 enhances ZO-1-associated intestinal epithelial barrier integrity via protein kinase C. *Gastroenterology*.

[75] Ey B., Eyking A., Gerken G., Podolsky D. K., Cario E. (2009). TLR2 mediates gap junctional intercellular communication through connexin-43 in intestinal epithelial barrier injury. *The Journal of Biological Chemistry*.

[76] Musil L. S., Goodenough D. A. (1991). Biochemical analysis of connexin 43 intracellular transport, phosphorylation, and assembly into gap junctional plaques. *The Journal of Cell Biology*.

[77] Martin F. J., Prince A. S. (2008). TLR2 regulates gap junction intercellular communication in airway cells. *Journal of Immunology*.

[78] Tran Van Nhieu G., Clair C., Bruzzone R., Mesnil M., Sansonetti P., Combettes L. (2003). Connexin-dependent inter-cellular communication increases invasion and dissemination of Shigella in epithelial cells. *Nature Cell Biology*.

[79] van Rijen H. V. M., van Kempen M. J. A., Postma S., Jongsma H. J. (1998). Tumour necrosis factor *α* alters the expression of connexin43, connexin40, and connexin37, in human umbilical vein endothelial cells. *Cytokine*.

[80] Kwak B. R., Hermans M. M., de Jonge H. R., Lohmann S. M., Jongsma H. J., Chanson M. (1995). Differential regulation of distinct types of gap junction channels by similar phosphorylating conditions. *Molecular Biology of the Cell*.

[81] Glukhov A. V., Fedorov V. V., Kalish P. W. (2012). Conduction remodeling in human end-stage nonischemic left ventricular cardiomyopathy. *Circulation*.

[82] Jain S. K., Schuessler R. B., Saffitz J. E. (2003). Mechanisms of delayed electrical uncoupling induced by ischemic preconditioning. *Circulation Research*.

[83] Vetter C., Zweifel M., Zuppinger C. (2010). Connexin 43 expression in human hypertrophied heart due to pressure and volume overload. *Physiological Research*.

[84] Agullo-Pascual E., Cerrone M., Delmar M. (2014). Arrhythmogenic cardiomyopathy and Brugada syndrome: diseases of the connexome. *FEBS Letters*.

[85] Asimaki A., Kapoor S., Plovie E. (2014). Identification of a new modulator of the intercalated disc in a zebrafish model of arrhythmogenic cardiomyopathy. *Science Translational Medicine*.

[86] Lorentz C. A., Liang Z., Meng M. (2017). Myosin light chain kinase knockout improves gut barrier function and confers a survival advantage in polymicrobial sepsis. *Molecular Medicine*.

[87] Zhang J., Yang G., Zhu Y., Peng X., Li T., Liu L. (2018). Relationship of Cx43 regulation of vascular permeability to osteopontin-tight junction protein pathway after sepsis in rats. *American Journal of Physiology. Regulatory, Integrative and Comparative Physiology*.

[88] Brooks T. A., Hawkins B. T., Huber J. D., Egleton R. D., Davis T. P. (2005). Chronic inflammatory pain leads to increased blood-brain barrier permeability and tight junction protein alterations. *American Journal of Physiology-Heart and Circulatory Physiology*.

[89] Cohen T. S., Gray Lawrence G., Margulies S. S. (2010). Cultured alveolar epithelial cells from septic rats mimic in vivo septic lung. *PLoS One*.

[90] Eadon M. T., Hack B. K., Xu C., Ko B., Toback F. G., Cunningham P. N. (2012). Endotoxemia alters tight junction gene and protein expression in the kidney. *American Journal of Physiology-Renal Physiology*.

[91] Sandbo N., Taurin S., Yau D., Kregel S., Mitchell R., Dulin N. (2007). Downregulation of smooth muscle *α*-actin expression by bacterial lipopolysaccharide. *Cardiovascular Research*.

