# Supplementary material for: Toll-Like Receptor-Mediated Cardiac Injury during Experimental Sepsis
Source: Mediators Inflamm. 2020 Jan 10;2020:6051983. doi: 10.1155/2020/6051983 (PMC7199613; doi:10.1155/2020/6051983)
Supplement: Supplementary Materials — Supplemental Figure 1: haematoxylin and eosin staining of the left ventricles of sham and CLP mice. Representative images were taken in 20x and 40x magnification. Mice received either sham treatment or CLP treatment for 24 h. [file 6051983.f1.pdf]

s

Supplemental Material

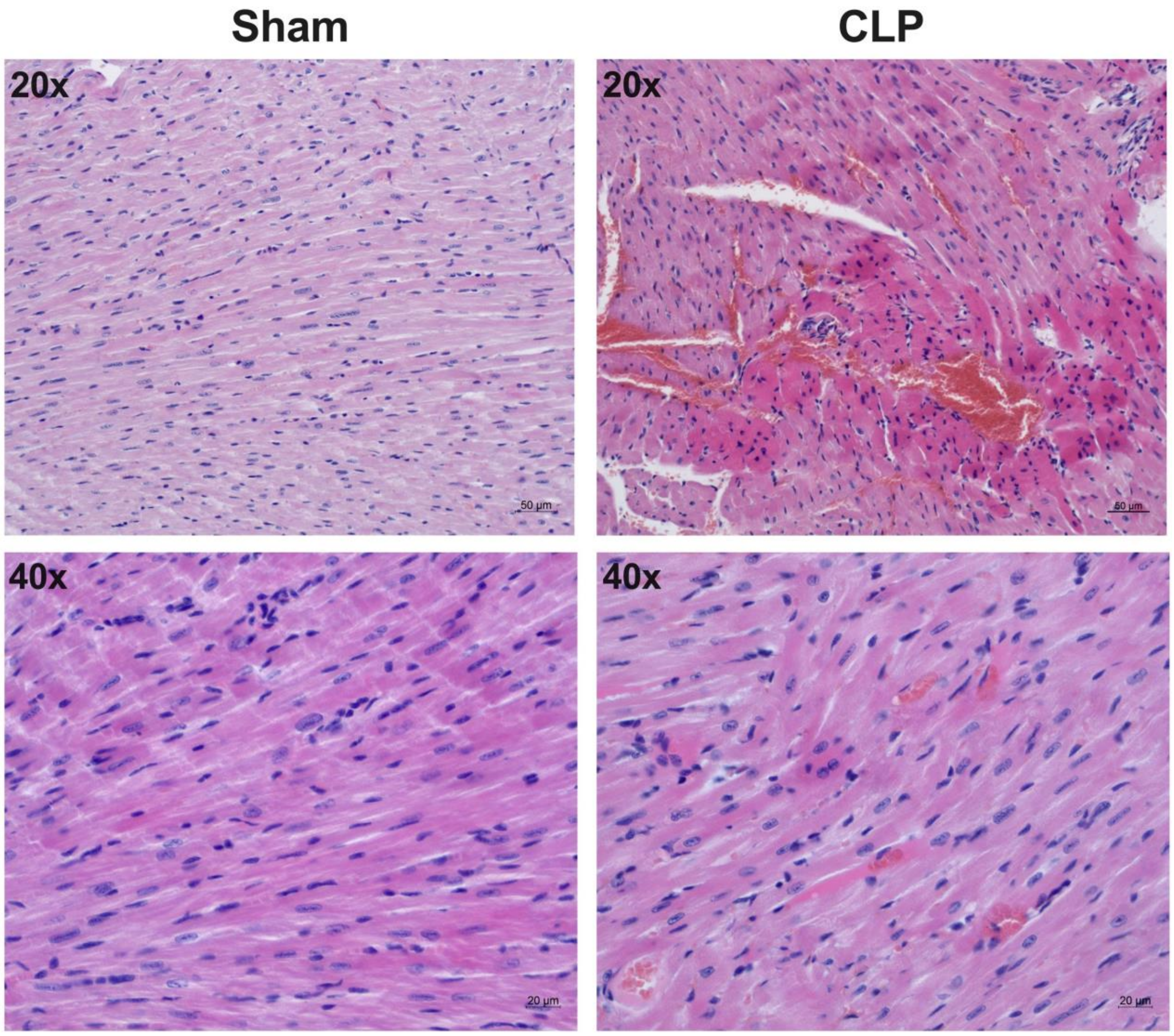

Supplemental Figure 1: Haematoxylin and Eosin staining of left ventricles of sham and CLP mice. Representative images were taken in 20x and 40x magnification. Mice received either sham-treatment or CLP treatment for 24 h.
